# Syphilitic hepatitis; a rare manifestation of a common disease 

**Published:** 2021

**Authors:** Flávio G. Pereira, Mariana S. Leal, Daniela Meireles, Susana Cavadas

**Affiliations:** *Internal Medicine Service, Centro Hospitalar do Baixo Vouga, Aveiro, Portugal*

**Keywords:** Acute Hepatitis, Secondary Syphilis, Syphilitic Hepatitis, Immunocompetent Patient, Sexually Transmitted Infection

## Abstract

Syphilis is a sexual transmitted disease caused by *Treponema pallidum* and an underdiagnosed and underreported cause of acute hepatitis. In recent years, reported cases of primary and secondary syphilis have been increasing, mostly in men who have sex with men. Clinical manifestations of syphilis are diverse, earning the name of “the great imitator” which can affect virtually any organ. Nonetheless, hepatic involvement is rare, but it can occur at any stage of the disease.

We present the case of a 41-year-old immunocompetent male, that presents to us with a cholestatic hepatitis and a diffuse erythematous rash with palmo-plantar affection. The patient had no history of primary syphilis. After throughout aetiologic study, he was diagnosed with syphilitic hepatitis and treated with intramuscular Benzathine benzylpenicillin, with the disappearance of the rash and normalization of liver enzymes after 3 months. We would like to highlight that this aetiology should be considered in patients with unexplained elevation of liver enzymes (mainly cholestatic enzymes) and an epidemiologic context of unsafe sexual exposure.

## Introduction

 Syphilis is a sexually transmitted disease caused by *Treponema pallidum*. Over the last years, its incidence has increased, mainly in people with risky sexual behaviour (1). It has been described as “the great imitator”, because it is a venereal disease with multiorgan involvement. Hepatic involvement is uncommon, but elevation of hepatic enzymes can be found in about 10% of the infected patients, whilst not having any other feature of hepatitis. Clinically relevant syphilitic hepatitis can occur at any phase of the disease. It is estimated that 3% of secondary syphilis cases evolve as hepatitis. Fulminant hepatitis or progression to cirrhosis is rare (2–5). 

## Case Report

**Figure 1:**
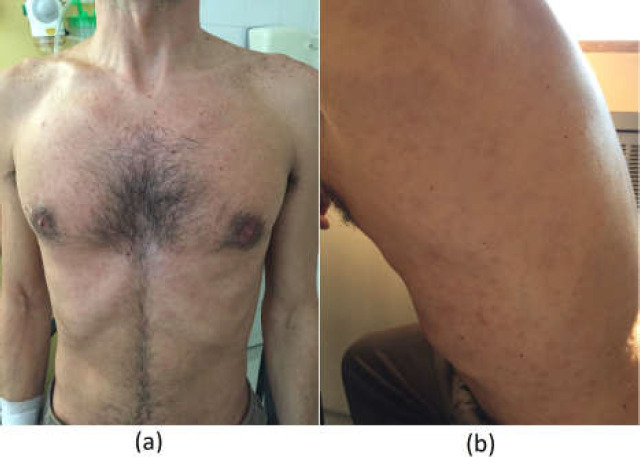
Photograph showing non-itchy, non-vesicular, macular, erythematous lesions of the anterior trunk (a) and left trunk (b)

We describe the case of a 41-years-old male that presents to our Emergency Room (ER) due to epigastric pain and anorexia. He is discharged on pantoprazole and sucralfate. Two days later, he develops a non-itchy rash of the trunk and limbs and discontinues the medication. One month later, he returns to the ER due to the persistence of severe epigastric pain, nausea and maintenance of the cutaneous lesions (Figure 1). He denied fever, loss of weight, hypersudoresis, jaundice, diarrhea or adenopathies. He had no significant personal or familiar background, besides history of unprotected sexual relations with multiple males. He denied taking any medication, alcohol, drugs or tobacco consumption. He also denied consumption of protein supplementation, non-prescribed medication or tea consumption.

On physical examination, he revealed non-itchy, non-vesicular, maculo-papular, erythematous lesions of the trunk and limbs, with palmo-plantar affection and pain to deep palpation of the epigastric region. Liver was palpable 1cm below costal border, with plain, regular and unpainful palpation. No genital, anal or oral lesions were found. No jaundice or others stigmas of hepatic disease were present.

Analytically, elevation of liver enzymes was found (aspartate aminotransferase 85U/L, alanine aminotransferase 178 U/L, alkaline phosphatase (ALP) 891U/L and gamma-glutamyl transferase 1844U/L), hyperbilirubinemia (1.57mg/dL) due to increase in the direct fraction (0.84mg/dL) and increased C Reactive Protein (4.55mg/dL). Prothrombin time and serum albumin were within normal values. Complete blood cell count was normal. Abdominal echography revealed globous liver, with a small increase in its size, without any other changes. We admitted patient to our ward for etiologic study of a cholestatic hepatitis. 

Virologic study revealed immunization by vaccination to HBV and immunization by contact to Cytomegalovirus. Antibodies against HAV, HCV, HEV, HIV, Toxoplasma gondii and Epstein-Barr were negative. Anti-nuclear, anti-smooth muscle, anti-mitochondrial and liver kidney microsomal antibodies were all negative. Serum plasmatic proteins electrophoresis, serum immunoglobulins, Alpha-1 antitrypsin and ceruloplasmina were within normal range. Total cholesterol was 191mg/dL and triglycerides 155mg/dL.

Magnetic resonance cholangiogram showed a slightly increased liver size (17cm in the longest axis in the longitudinal plane), with homogeneous structure, without steatosic infiltration or any other stigma of chronic hepatic disease. No intra or extra hepatic biliary ducts stenosis or dilation were found. Given severe epigastric pain, upper digestive endoscopy was requested. No changes were found in endoscopy.

**Figure 2:**
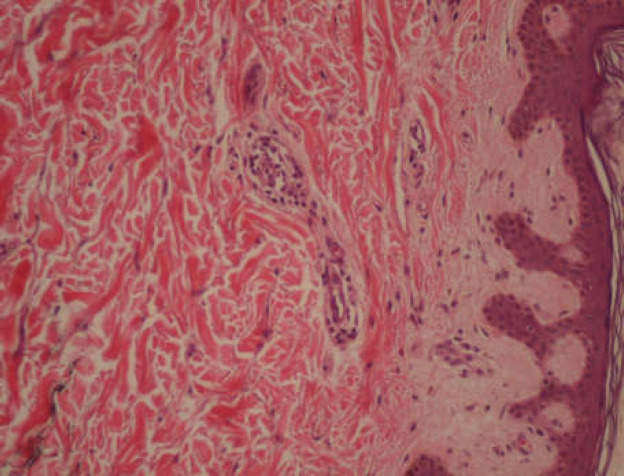
Histopathological findings – superficial perivascular infiltrate of lymphomonocytic round cells (haematoxylin-eosin x200) (2)

Given the sexual history of our patient and the clinical presentation, syphilis test was performed. VDRL test was reactive (64 dilutions), with positive microhemagglutination assay test (>5120 dilutions). Skin lesions were biopsied which revealed superficial perivascular infiltrate of lymphomonocytic round cells, with rare plasmocytic cells. This result was compatible with syphilitic roseola (Figure 2).

**Figure 3:**
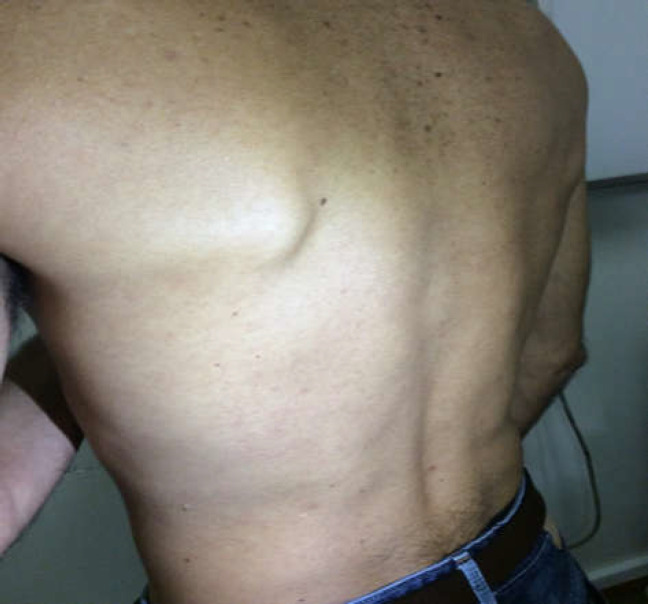
Photograph 3 weeks after treatment with Benzathine benzylpenicillin

Secondary syphilis was assumed with syphilitic hepatitis. Patient was medicated with a unique dose of intramuscular Benzathine benzylpenicillin (2.4 million units). After treatment, he presented favourable clinic and analytic evolution and was discharged and oriented to our outpatient clinic. Three weeks later, in an office visit, all skin lesions had disappeared (Figure 3). Ten weeks after treatment, liver enzymes were within normal range (Table 1). Nine months after treatment, VDRL was non-reactive and patient continued to present normal liver enzymes and negative HIV test.

## Discussion

The etiologic agent of syphilis, *Treponema pallidum*, can infect any organ. Cutaneous, genital and central nervous system involvements are exhaustively described (6). However, syphilis can have atypical presentations, such as hepatitis. In recent years, most cases of syphilis have been reported in young males who have unprotected sex with males (around 67% of recently reported primary or secondary syphilis) (7–9).

**Table 1:** Analytical evolution from the initial presentation till 9 months after treatment

	13/01	16/01	23/01	07/02	05/04	24/10
AST (U/L)	85	52	45	62	34	27
ALT (U/L)	178	106	87	123	61	31
ALP (U/L)	891	665	575	251	91	71
G-GT (U/L)	1844	1489	1319	577	79	27
TB (mg/dL)	1.57	1.26	1.17	0.87		1.28
PT (seconds)	11.80	11.60	12.10			
VDRL	Reactive (64 dilutions)					Non-reactive

Note: aspartate aminotransferase (AST), alanine aminotransferase (ALT), alkaline phosphatase (ALP); gamma-glutamyl transferase (G-GT), Total Bilirubin (TB), Prothrombin time (PT).

In almost all cases of syphilitic hepatitis, it is common to find a disproportional increase in ALP, when compared to the increase in aminotransferases. (9). Histopathological examination is non-specific. Lymphocytic infiltrate of portal spaces with focal necrosis of peri-portal hepatocytes can be found (8–10). Pathophysiology of hepatic syphilitic lesion is unknown, but it is thought to have been caused by pericholangiolar inflammation present in these situations (4,7). Multisystemic involvement can be justified by an immune-mediated mechanism, such as the host inflammatory response to the infection. Microorganism identification in hepatic biopsy is pathognomonic, yet rarely achievable (11).

In Mullick et al. (2004), the proposed diagnostic criteria for syphilitic hepatitis are as follows: (i) abnormal liver enzyme levels indicating hepatic involvement; (ii) serological evidence for syphilis, with a positive RPR titer and a reactive FTA-Abs result or microhemagglutination assay result positive for T. pallidum (MHA-TP) in conjunction with an acute clinical presentation consistent with secondary syphilis; (iii) exclusion of alternative causes of hepatic damage, such as acute viral hepatitis, use of medication, malignancy, or opportunistic infection; and (iv) improvements in liver enzyme levels following appropriate antimicrobial therapy (7).

In our case, the presence of exanthema and the exclusion of other causes of cholestatic hepatitis raised the suspicion of secondary syphilis. This diagnosis was confirmed by VDRL, MHA-TP and the skin biopsy result. There was no history of primary syphilis. It is important to highlight that genital syphilis lesion is most often unpainful and most of the times does not need any treatment to heal and may not be diagnosed. (10) In the end, the favourable clinical and analytical response to the administration of Benzathine benzylpenicillin confirmed the diagnosis of syphilitic hepatitis, according to Mullick’s diagnostic criteria (7).

With this case, the authors intend to highlight that syphilis (“the great imitator”) should be considered for the differential diagnosis of acute or chronic cholestatic hepatitis. This is particularly important in sexually active patients, because it is reversibly and easily treatable, although it can evolve to fulminant hepatitis (12). This high index of suspicion should include immunocompetent patients.

## Funding Sources

The authors declare that there were no external financial sources for this study.

## Conflict of interests

The authors declare that they have no conflict of interest.
